# Failure of syngeneic bone marrow cells to protect against MC-induced lymphoma in dba-2 mice.

**DOI:** 10.1038/bjc.1968.69

**Published:** 1968-09

**Authors:** L. Chen, I. Berenblum


### FAILURE OF SYNGENEIC BONE MARROW CELLS TO PROTECT AGAINST MC-INDUCED LYMPHOMA IN dba/2 MICE

LOUISE CHEN AND I. BERENBLUM

*From the Department of Experimental Biology, Weizmann Institute of Science, Rehovoth, Israel*

Received for publication May 7, 1968

IT has been shown long ago that shielding the bone marrow (Kaplan and Brown, 1951) or spleen (Lorenz *et al.*, 1953) of mice during exposure to whole-body X-irradiation, or injection of syngeneic bone marrow cells after whole-body irradiation (Kaplan *et al.*, 1953), reduces the incidence of thymic lymphomas resulting from the irradiation. How the effect is brought about has never been satisfactorily explained. There is presumably some essential factor in the normal hematopoietic system which irradiation is able to depress. Whether this inhibitory process affects a specific stage in radiation leukaemogenesis, or whether it is concerned with the leukaemogenic process as such, irrespective of the nature of the leukaemogenic agent, has not so far been fully established. Indirect evidence would suggest, however, that the former (i.e. a specific anti-radiation effect) is the more likely explanation.

It is known, for instance, that bone marrow cells of C3H mice, though acceptable to tolerant AKR mice, fail to inhibit the spontaneous development of thymic lymphomas in the latter strain (Miller, 1960). In the case of leukaemogenesis by the combined action of X-irradiation and estrogen injection, the estrogen almost nullified the protective effect of thigh shielding (Toch *et al.*, 1956). In previous experiments in this laboratory (Berenblum *et al.*, 1966), it has also been shown that normal syngeneic bone marrow cells fail to inhibit urethane leukaemogenesis in newborn C57BL mice. The results with cell-free extracts of sheep spleen (a protein component—RLP—recently characterized as 19S alpha-2 globulin (Berenblum *et al.*, 1968), capable of inhibiting radiation leukaemogenesis in C57BL mice, but not of the spontaneous disease in AKR mice (Berenblum *et al.*, 1967), also supports the view that the inhibition is strictly anti-radiation in its mode of action.

A critical factor in the inhibition of radiation leukaemogenesis by syngeneic bone marrow cells is the time of injection of the cells in relation to the irradiation. For effective inhibition, the cell suspension must be injected within a few hours after the last irradiation (Kaplan *et al.*, 1955). In the urethane experiment quoted above (Berenblum *et al.*, 1966), the bone marrow cell suspension was injected 2–4 days after the urethane injection, in order to avoid the possibility of the bone marrow cells being damaged by the urethane *in vivo*. The interpretation of the results (failure of the bone marrow cells to interfere with urethane leukaemogenesis), might therefore seem less convincing than would be desired.

The present series of experiments, using 3-methylcholanthrene (MC) as the chemical leukaemogenic agent, and dba/2 mice as test animals, were designed to overcome these limitations.

### MATERIALS AND METHODS

### *Animals*

The dba/2 mice used in these experiments were obtained from the Jackson Laboratory, Bar Harbor, Maine. They were kept for a week or more before use, to acclimatize them to the conditions of the laboratory, and so that they should reach the required age for the respective experiments. The animals were housed in an air-conditioned room at 21–24° C., fed Purina Laboratory Chow, supplemented occasionally with barley and sunflower seeds, and provided with tap water *ad libitum.*

### *X-rays*

The X-irradiation was performed with a General Electric Maximar 250-III machine. The physical factors were: 250 kv., 15 mA., with 1 mm. Al and 0·5 mm. Cu filters: dose rate 61 R/minute for the 200 R and 800 R exposures, and 37 R/ minute for the 150 R exposure.

In Expt. I, male and female mice, 6 weeks old at the start of the experiment, received 12 applications of 3-MC to the skin, under the following conditions: a single drop of 0·25 per cent MC in acetone (containing approximately 1 $\mu$g. MC) was applied 3 times a week to different areas of skin in rotation, thereby minimizing the local development of tumours. Previous removal of hair was performed by means of an electric clipper. The mice were then divided into 2 groups: Group 1 received no further treatment; Group 2 was given, in addition, 3 intravenous injections of bone marrow cells 2–3 hours after the third, sixth and twelfth MC application. The bone marrow cells were in the form of a suspension containing $15 \times 10^6$ cells per injection, prepared according to the procedure described previously (Berenblum *et al.*, 1966). A third group of mice, of both sexes and of the same age, was kept without any treatment, to provide information about the spontaneous incidence of leukaemia in the strain under the conditions of this laboratory.

Two additional experiments were set up to test the effectiveness of the bone marrow suspensions derived from MC-treated mice:

To test the ability of the cell suspension to repair radiation damage to the thymus (Expt. II), 6-week-old male mice were given 2 exposures of 200 R each at 7-day intervals, and then divided as follows: One group received $15 \times 10^6$ syngeneic bone marrow cells from mice pretreated with 12 applications of MC. The bone marrow cells were taken from the donors 2–3 hours after the last MC treatment. A second group was injected with an equivalent number of bone marrow cells from normal untreated mice. In both groups the bone marrow cells were injected 2–3 hours after the second irradiation. In the third group, no bone marrow injection was given. A fourth group was kept without any treatment. The animals were killed 21 days after the last irradiation, and thymic weights were determined.

To test for the ability of bone marrow from pretreated animals to protect lethally-irradiated mice (Expt. III), male mice, 3–4 months old, were given a single lethal dose of 800 R of total-body X-irradiation. The irradiated mice were divided into 3 groups, and treated similarly to those in the previous experiment. The first group received bone marrow from MC-treated mice; the second group received bone marrow from untreated mice; and the third group served as irradiated, uninjected control mice.

The effectiveness of bone marrow with respect to survival was determined by the number of survivors 60 days after irradiation.

In order to compare MC-induced leukaemogenesis with radiation-induced leukaemogenesis in dba/2 mice, the following experiment (IV) was also included:

Two groups of mice of both sexes were submitted to 4 weekly exposures of 150 R each of total-body X-irradiation (totalling 600 R). Approximately 3–4 hours after the last irradiation, the mice of Group 1 received one intravenous injection of a normal bone marrow cell suspension, containing $15 \times 10^6$ cells per injection. The mice of Group 2 served as uninjected controls.

### RESULTS

The results of Expt. I are summarized in Table I. Twelve applications of

TABLE I.—*Incidence of Lymphatic Lymphomas in dba/2 Mice Given MC and Syngeneic Bone Marrow*

| Group | Treatment | Sex | No. of mice used | No. of lymphomas/ effective total* | Incidence of lymphomas (%) | Average latent period (weeks) |
|---|---|---|---|---|---|---|
| 1 | MC | ♀ | 22 | 13/22 | 59 | 25·3 |
| | | ♂ | 35 | 6/32 | 19 | 42·6 |
| | | Total | 57 | 19/54 | 35 | 30·8 |
| 2 | MC + bone marrow | ♀ | 22 | 11/21 | 52 | 30·9 |
| | | ♂ | 31 | 5/27 | 19 | 27·2 |
| | | Total | 53 | 16/48 | 33 | 29·7 |
| 3 | Untreated | ♀ | 5 | 0/5 | 0 | — |
| | | ♂ | 18 | 0/18 | 0 | — |
| | | Total | 23 | 0/23 | 0 | — |

* Effective total = number of survivors at time of appearance of first leukaemia.

MC alone yielded a 35 per cent incidence of leukaemia at a mean age of 30·8 weeks; while such treatment together with 3 injections of normal syngeneic bone marrow yielded a similar incidence of leukaemia (33 per cent) with a mean latent period of 29·7 weeks. A remarkable sex difference was observed in both groups, the females developing 3 times more leukaemia than the males. Twenty-three untreated controls of both sexes remained free from leukaemia during a period of observation of 55 weeks.

In the autopsy findings, there was swelling of the liver and spleen, and great enlargement of all the lymph nodes. The enlargement of the thymus was relatively slight. Histologically, neoplastic tissue was of stem cell or lymphoblastic type.

In Table II are presented the results of Expt. II, in which equal numbers of bone marrow cells from normal and MC-treated donors were tested for their ability to promote thymic regeneration in sublethally irradiated mice. The mean thymic weight of the untreated dba/2 mice was $30{\cdot}7 \pm 4{\cdot}3$ mg. Twenty-one days after 2 exposures to irradiation, the mean thymic weight dropped to $16{\cdot}6 \pm 2{\cdot}9$ mg. Bone marrow from animals treated with MC, when injected to irradiated mice, led to regeneration of the damaged thymus, as manifested by increasing thymus weight to $37{\cdot}3 \pm 3{\cdot}4$ mg., somewhat similar to the results with bone marrow from normal donors—$40{\cdot}2 \pm 4{\cdot}8$ mg. The mean thymic weight of irradiated mice without added treatment, and of those which received bone marrow from MC-treated animals, differed significantly ($P < 0{\cdot}001$).

TABLE II.—*Effect of Bone Marrow Pretreated with MC on Thymic Recovery in Sublethally Irradiated Mice*

| Group | Treatment | No. of recipients | Mean thymic weight ± standard deviation (mg) | $t$* |
|---|---|---|---|---|
| 1 | 200 R × 2 followed by bone marrow from MC pretreated mice | 17 | 37·3±3·4 | <0·001 |
| 2 | 200 R × 2 followed by normal bone marrow | 16 | 40·2±4·8 | |
| 3 | 200 R × 2 | 15 | 16·6±2·9 | |
| 4 | None | 15 | 30·7±4·3 | |

* $t$ = Significance of the difference between the mean thymic weight of experimental group 1 and control group 3 as evaluated by the $t$ test.

TABLE III.—*Effect of Bone Marrow Pretreated with MC on the Survival of Lethally Irradiated Mice*

| Treatment | 60-day survival rate | Per cent survival |
|---|---|---|
| 800 R followed by bone marrow from MC pretreated mice | 15/16 | 94 |
| 800 R followed by normal bone marrow | 13/15 | 87 |
| 800 R | 0/16 | 0 |

In Expt. III, it was found that MC did not diminish the ability of bone marrow to protect lethally-irradiated animals. Sixty-day survival was 94 per cent in the mice irradiated with 800 R and injected with bone marrow from MC-treated mice, and 87 per cent in those irradiated and injected with normal bone marrow. All the irradiated control animals died at day 13.

TABLE IV.—*Incidence of Lymphatic Lymphomas in dba/2 Mice Irradiated and Injected with Syngeneic Bone Marrow*

| Group | Treatment | Sex | No. of mice used | No. of lymphomas/ effective total* | Incidence of lymphomas (%) | Average latent period (weeks) |
|---|---|---|---|---|---|---|
| 1 | 150 R × 4 | ♀ | 54 | 26/51 | 50 | 43·1 |
| | | ♂ | 35 | 5/32 | 17 | 41·4 |
| | | Total | 89 | 31/83 | 37 | 42·7 |
| 2 | 150 R × 4 followed by normal bone marrow | ♀ | 11 | 3/11 | 27 | 50·3 |
| | | ♂ | 30 | 0/27 | 0 | — |
| | | Total | 41 | 3/38 | 8 | 50·3 |

* Effective total = number of survivors at time of appearance of first leukaemia.

The results of Expt. IV showed that dba/2 mice irradiated with 150 R × 4 developed an incidence of 37 per cent lymphomas at a mean latent period of 42·7 weeks, and showed the same significant sex difference as was found in the previous experiment: 50 per cent lymphomas in females and 17 per cent in males. Normal bone marrow inhibited lymphoma induction, decreasing the total incidence to

8 per cent after a latent period of 50·3 weeks. An incidence of 27 per cent lymphomas was still found in females, compared with 0 per cent in males.

### DISCUSSION

From the experiments presented here, it may be concluded that synegenic bone marrow cells, injected into MC-treated dba/2 mice, fail to inhibit leukaemogenesis (lymphoma induction), though they do inhibit radiation leukaemogenesis, as in the case of C57BL mice.

Previous studies (Kaplan and Brown, 1957) have shown that whole-body X-irradiation can destroy hematopoietic cells, and the suggestion has been put forward that this might play an important role in leukaemogenesis. On the other hand, the present results do not indicate that chemical leukaemogenesis (with MC) causes significant damage to the hematopoietic system under the conditions used, since bone marrow suspensions from such treated animals were found to be as effective as normal bone marrow suspensions in (a) repairing acute radiation damage in the thymus, and (b) preventing the lethal action of higher doses of whole-body irradiation.

Earlier evidence (Toch *et al.*, 1956; Miller, 1960; Berenblum *et al.*, 1966), referred to in the Introduction, already indicated that inhibition of leukaemogenesis by bone marrow cells is probably specific for radiation leukaemogenesis, and not operative in spontaneous leukaemia development or in chemical leukaemogenesis. The present results fully support this conclusion. This does not necessarily mean that the mechanisms of leukaemogenesis in spontaneous, radiation-induced and chemically-induced leukaemia, are different, but merely indicates that radiation leukaemogenesis is a more elaborate process, and that one of the complicating factors—some specific damage to the hematopoietic system—can be counteracted by injection of normal syngenic bone marrow cells. The effect, in short, is on recovery from radiation damage, as distinct from on the sequence of biological changes in the development of leukaemia. It is interesting to note in this connection that injection of bone marrow cells, which is effective in protecting against lethal doses of radiation, does not prevent the mortality resulting from lethal doses of a chemical leukaemogen (Congdon *et al.*, 1964).

Following earlier conflicting evidence (see Miller, 1961), recent results of Haran-Ghera and Peled (1967) support the view that the protective action of hematopoietic cells is associated with a restoration of the depressed immune response resulting from the irradiation. Experiments are now in progress here to test whether chemical carcinogens, employed for leukaemogenesis, also affect the immunological potency of bone marrow cells, and if so, whether this could also play a critical role in leukaemogenesis.

Female mice are generally more susceptible than male mice to spontaneous lymphoma development (McEndy *et al.*, 1944) or to lymphoma induction by X-irradiation (Kaplan and Brown, 1952), though in the case of lymphoma induction by MC skin painting in dba/2 mice, little or no sex difference in incidence was reported by previous workers (Andervont and Dunn, 1953; Kirschbaum *et al.*, 1955). Our results with MC skin painting in dba/2 mice did show a striking sex difference, the incidence in females being about 3 times that in males. Estrogens are known to enhance the leukaemogenic action of MC in dba/2 mice, which are, in fact, susceptible to this chemical alone (Kirschbaum *et al.*, 1953). Since female mice have a higher endogenous estrogen than male mice, a lower dose of MC might be effective for leukaemogenesis in females than in males, while this difference might be masked when excessive doses of MC are given. This could explain the difference in sex ratio between our results with MC and those reported by others (Kirschbaum and Mixer, 1947; Kirschbaum *et al.*, 1955).

### SUMMARY

Injections of a suspension of syngeneic bone marrow cells to methylcholanthrene-treated adult dba/2 mice failed to inhibit the induction of leukaemia (lymphoma), in contrast to its inhibiting effect on radiation-induced leukaemia in the same strain. That the MC did not itself destroy the injected bone marrow cells was checked by independent tests. The results suggest a specific anti-radiation effect of bone marrow cells rather than a general antileukaemic influence.

The incidence of lymphomas following 12 applications of MC was about 3 times higher in females than in males. A possible interpretation of these findings is discussed.

This investigation was supported by U.S. Public Health Service Research Grant No. CA-05455 from the National Cancer Institute.

We are indebted to Mrs. Yehudith Greenberg for valuable technical assistance.

### REFERENCES

ANDERVONT, H. B. AND DUNN, T. B.—(1953) *J. natn. Cancer Inst.*, **14**, 329.
BERENBLUM, L., BOIATO, L. AND TRAININ, N.—(1966) *Cancer Res.*, **26**, 357.
BERENBLUM, I., BURGER, M. AND KNYSZYNSKI, A.—(1968) *Nature, Lond.*, **217**, 857.
BERENBLUM, I., TRAININ, N., CIVIDALLI, G. BOIATO-CHEN, L. AND KNYSZYNSKI, A.—(1967) *Tumori*, **53**, 5.
CONGDON, C. C., DOHERTY, D. G. AND HACKER, F.—(1964) *Blood*, **24**, 661.
HARAN-GHERA, N. AND PELED, A.—(1967) *Br. J. Cancer*, **21**, 730.
KAPLAN, H. S. AND BROWN, M. B.—(1951) *J. natn. Cancer Inst.*, **12**, 427.—(1952) *J. natn. Cancer Inst.*, **13**, 185.—(1957) In: 'The Leukemias: Etiology, Pathophysiology and Treatment'. Henry Ford Hospital Internat. Symp. New York and London (Academic Press), pp. 163–175.
KAPLAN, H. S., BROWN, M. B. AND PAULL, J.—(1953) *J. natn. Cancer Inst.*, **14**, 303.
KAPLAN, H. S., MOSES, L. E., BROWN, M. B., NAGAREDA, C. S. AND HIRSCH, B. B.—(1955) *J. natn. Cancer Inst.*, **15**, 975.
KIRSCHBAUM, A., LIEBELT, A. G. AND FALLS, N. G.—(1955) *Cancer Res.*, **15**, 685.
KIRSCHBAUM, A. AND MIXER, H.—(1947) *J. Lab. clin. Med.*, **32**, 720.
KIRSCHBAUM, A., SHAPIRO, J. R. AND MIXER, H. W.—(1953) *Cancer Res.*, **13**, 262.
LORENZ, E., CONGDON, C. C. AND UPHOFF, D. E.—(1953) *J. natn. Cancer Inst.*, **14**, 291.
MCENDY, D. P., BOON, M. C. AND FURTH, J.—(1944) *Cancer Res.*, **4**, 377.
MILLER, J. F. A. P.—(1960) *Br. J. Cancer*, **14**, 244.—(1961) *Adv. Cancer Res.*, **6**, 292.
TOCH, P., HIRSCH, B. B., BROWN, M. B., NAGAREDA, C. S. AND KAPLAN, H. S.—(1956) *Cancer Res.*, **16**, 890.


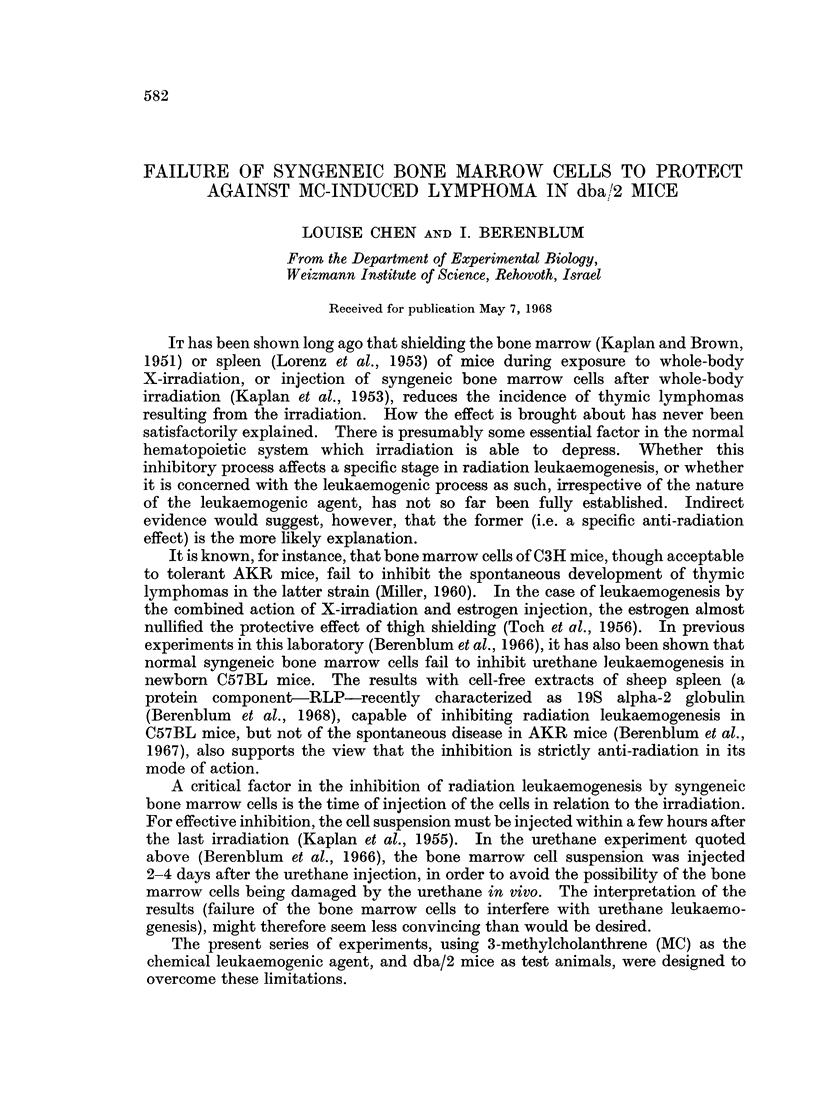

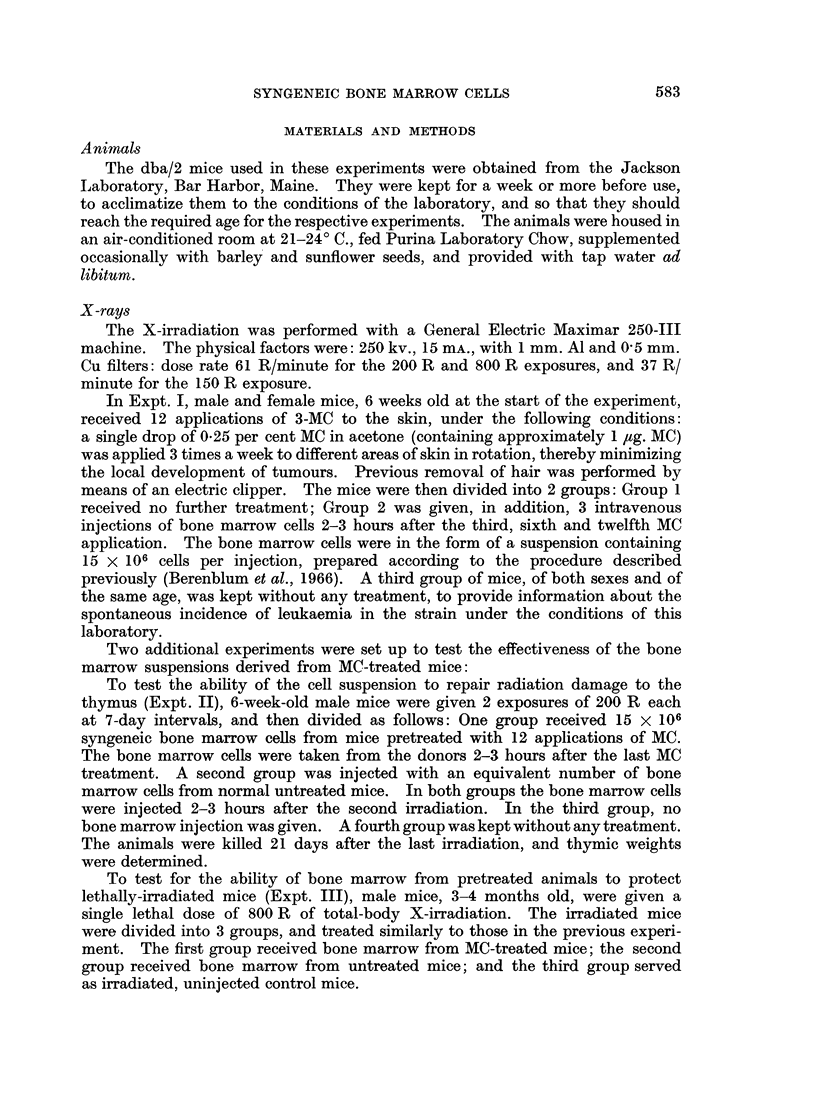

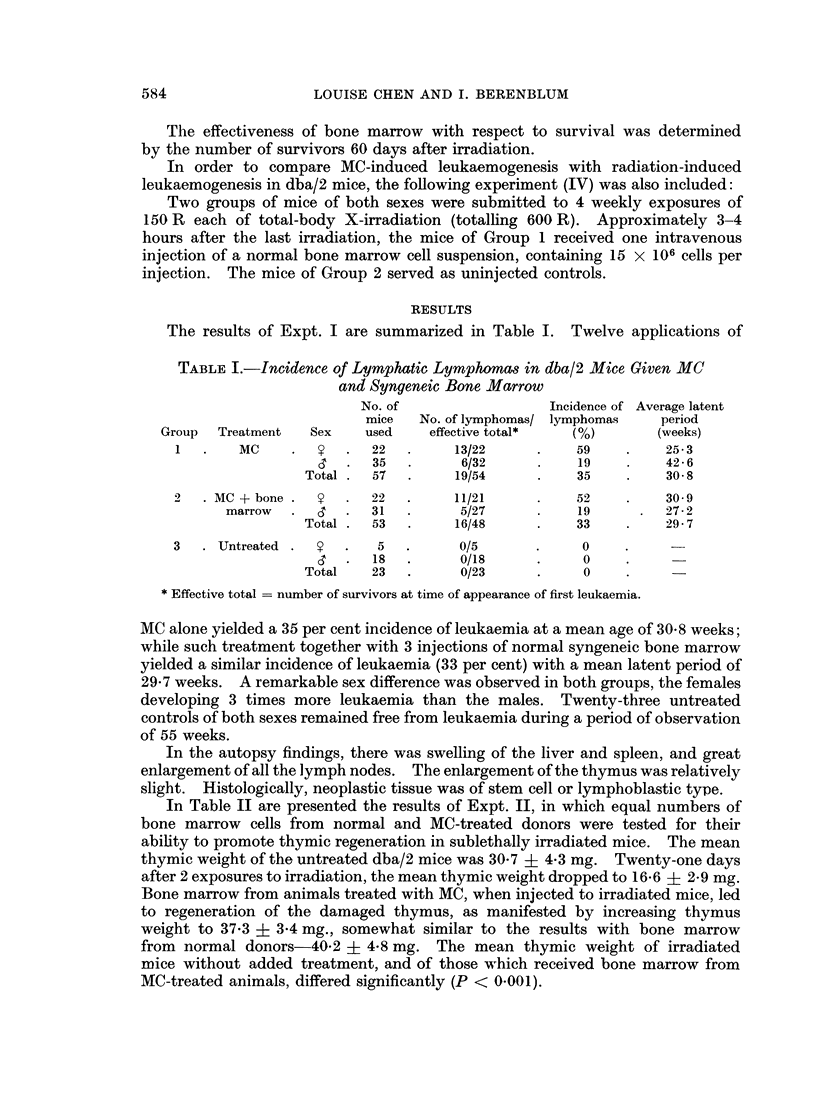

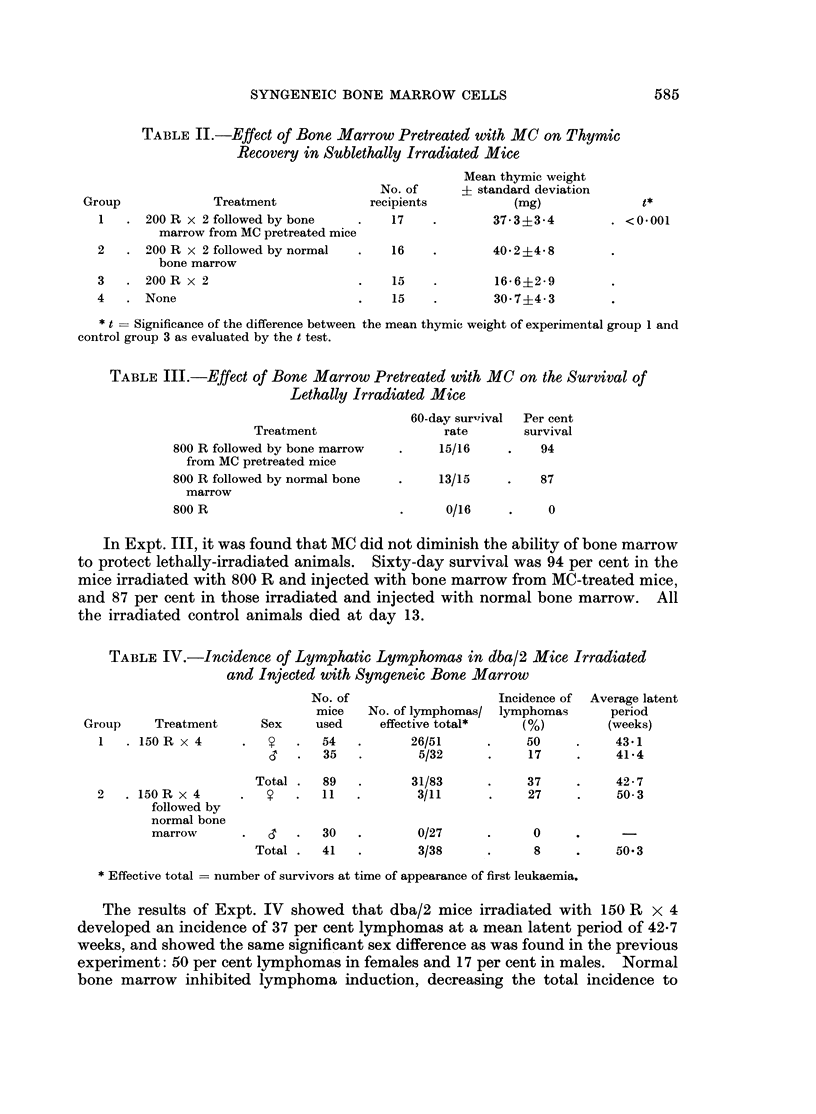

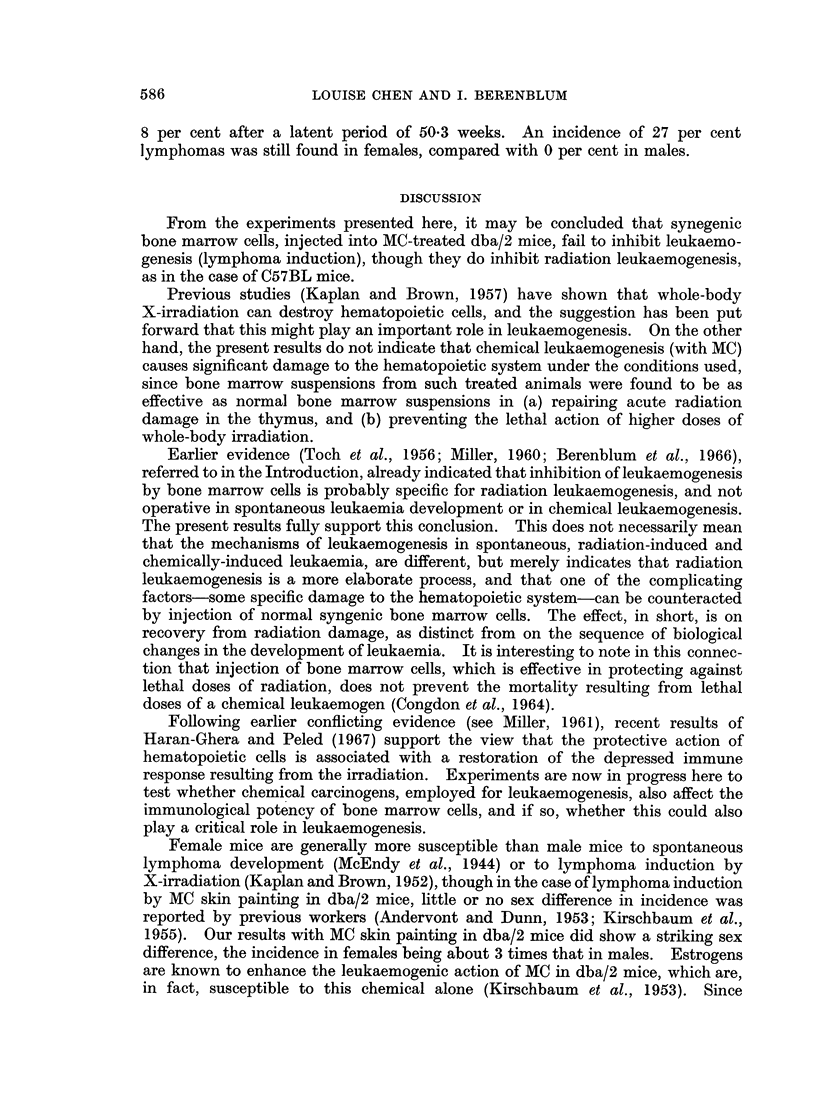

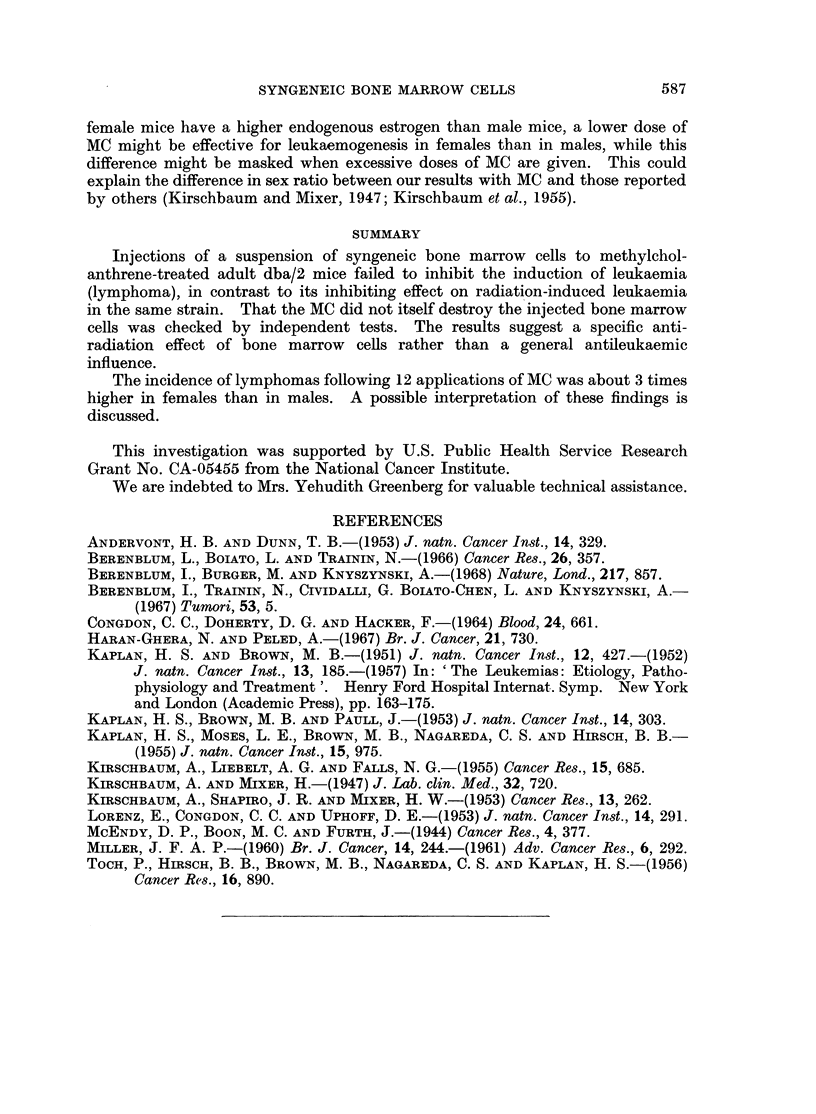


## References

[OCR_00461] BROWN M. B., HIRSCH B. B., KAPLAN H. S., NAGAREDA C. S., TOCH P. (1956). Lymphoid tumor incidence in mice treated with estrogen and x-radiation.. Cancer Res.

[OCR_00433] Berenblum I., Burger M., Knyszynski A. (1968). Regeneration of bone marrow cells and thymus induced by 19S alpha-2 globulin in irradiated mice.. Nature.

[OCR_00442] KAPLAN H. S., BROWN M. B. (1951). Further observations on inhibition of lymphoid tumor development by shielding and partial-body irradiation of mice.. J Natl Cancer Inst.

[OCR_00450] KAPLAN H. S., MOSES L. E., BROWN M. B., NAGAREDA C. S., HIRSCH B. B. (1955). The time factor in inhibition of lymphoid-tumor development by injection of marrow cell suspensions into irradiated C57BL mice.. J Natl Cancer Inst.

[OCR_00452] KIRSCHBAUM A., LIEBELT A. G., FALLS N. G. (1955). Influence of gonadectomy and androgenic hormone on the induction of leukemia by methylcholanthrene in DBA/2 mice.. Cancer Res.

[OCR_00453] KIRSCHBAUM A., SHAPIRO J. R., MIXER H. W. (1953). Synergistic action of leukemogenic agents.. Cancer Res.

[OCR_00457] LORENZ E., CONGDON C. C., UPHOFF D. (1953). Prevention of irradiation-induced lymphoid tumors in C57BL mice by spleen protection.. J Natl Cancer Inst.

